# Tumor refractoriness to anti-VEGF therapy

**DOI:** 10.18632/oncotarget.8694

**Published:** 2016-04-11

**Authors:** Domenico Ribatti

**Affiliations:** ^1^ Department of Basic Medical Sciences, Neurosciences and Sensory Organs, University of Bari Medical School, Bari, Italy; ^2^ National Cancer Institute “Giovanni Paolo II”, Bari, Italy

**Keywords:** angiogenesis, anti-angiogenesis, resistance, tumor growth, VEGF

## Abstract

Vascular endothelial growth factor (VEGF) has been identified as the most potent cytokine involved in tumor angiogenesis and metastasis formation. Clinical results of anti-angiogenic therapies targeting VEGF and its receptors are very modest, resulting in a moderate improvement of overall survival. The clinical outcome is associated with the development of resistance and the increased risk of invasion and metastasis. In this article, I have analyzed the principal mechanisms of resistance to VEGF pathway inhibitors, including normalization of tumor blood vessels, hypoxia, recruitment of inflammatory cells and immature myeloid cells, alternative mechanisms of tumor vessel formation, genomic instability of tumor endothelial cells. In this context, the concept and strategies of anti-angiogenic therapies should be extensively re-considered and re-evaluated. In particular, rational combinations of anti-angiogenic agents based on pharmacokinetic and pharmacodynamics data are needed to overcome resistance and it is extremely important to determine the optimal duration and scheduling of anti-VEGF agents.

## INTRODUCTION

The introduction of chemotherapy in 1950-60 resulted in the development of curative therapeutic interventions for patients with solid and hematologic tumors. While chemotherapy in the neo-adjuvant setting has typically resulted in improved survival following surgical intervention, similar benefits with anti-angiogenic therapy remain largely untested.

Tumor cells and tumor-associated stroma are sources of vascular endothelial growth factor (VEGF), which is responsible of vascular proliferation and altered permeability of newly formed vessels [1]. Several strategies to inhibit VEGF-VEGF receptors (VEGFRs) signaling pathway for the treatment of cancer have been explored. In addition to monoclonal antibodies, alternative approaches of inhibiting VEGFRs by using anti-VEGFRs small receptor tyrosine kinase inhibitors (TKIs) have been explored (Table 1).

Even if the majority of pre-clinical studies have shown that the growth of all experimental tumors can be effectively inhibited by various anti-angiogenic agents, the clinical benefits of these treatments are relatively modest, because the drugs merely slow down tumor progression and prolong survival by only a few more months [2-4]. In multiple randomized phase III clinical trials, bevacizumab conferred a survival benefit only when administered in combination with chemotherapy. Examples of metastatic cancers where anti-angiogenic therapy failed to make a significant impact on overall survival include breast, melanoma, pancreatic and prostate.

Moreover, as it has been demonstrated in animal models, anti-angiogenic therapy caused marked regression of normal microvessels in endocrine glands (thyroid, adrenal glands, pancreatic islets) and in the liver, kidney and gastrointestinal wall [5, 6], and angiogenesis inhibitors can decrease the delivery of cytotoxic drugs [7].

It is important to note that when VEGF-targeted therapies are discontinued, the tumor vasculature ca become rapidly re-established [8]. These data suggest that prolonged use of VEGF-targeted therapy is necessary to achieve maximal therapeutic effect. An observational study has shown that continuation of bevacizumab treatment beyond progression was associated with greater benefit in terms of overall survival [9].

Intrinsic resistance is characterized by inefficacy of tumor treatment with anti-angiogenic anti-VEGF, fusion proteins that trap VEGF [10], and anti-VEGFRs small receptor TKIs [11-13]. TKIs target VEGFR-1, -2, -3 signaling pathways and other members of the platelet-derived growth factor (PDGF) receptor and fibroblast growth factor (FGF) receptor families. In this context, TKIs inhibitors would be more effective than antibody-based therapy that solely target the VEGF pathway. Nevertheless, in acquired resistance, alternative mechanisms lead to activation of angiogenesis even when the target of the drug remains inhibited [14, 15].

Trials that have combined monoclonal antibodies and TKIs have given rise to an increase in the side effects profile, including hypertension, gastrointestinal perforation, hemorrhages, proteinuria, anemia, leucopenia, and thrombocytopenia.

**Table 1:** Principal anti-VEGF molecules approved by FDA for anti-angiogenic cancer treatment

Name	Target
Bevacizumab	VEGF-A
Aflibercept	VEGF-A
Axitinib	VEGFRs
Cabozantinib	VEGFRs
Regorafenib	VEGFRs
Sorafenib	VEGFRs
Sunitinib	VEGFRs
Vandetanib	VEGFRs
Pazopanib	VEGFRs

## NORMALIZATION OF TUMOR BLOOD VESSELS

In 2001, Rakesh Jain introduced the concept of “normalization” of tumor blood vessels by anti-angiogenic molecules [16]. VEGF inhibition could temporarily restore or normalize the function of tumor-associated vasculature, decreasing vascular permeability in conjunction with restoration of sustained pressure gradients, thereby enhancing systemic delivery of oxygen or perfusion of cytotoxic agents to intratumoral sites. Moreover, abrogation of VEGF signaling increases collagenase IV activity, leading to restoration of normal basement membrane, which generally in tumors has an abnormally thickness.

Moreover, tumor vascular normalization is accompanied by increased pericyte coverage. Pericyte deficiency could be partly responsible for vessel abnormalities in tumor blood vessels [17] and partial dissociation of pericytes [18, 19] contribute to increased tumor vascular permeability.

Anti-angiogenic refractory tumors contained blood vessels with a investment of pericytes expressing alpha smooth muscle actin (α-SMA) [20]. Pericyte coverage promote resistance through direct support or paracrine interactions with endothelial cells and tumor vessels covered by pericytes are less sensitive to VEGF blockade [21]. Pericytes can activate compensatory PDGFR-mediated pro-angiogenic signaling in anti-VEGF therapy [22]. Bergers et al. showed that combined treatment or pre-treatment with anti-PDGF-B/PDGFBR-β reducing pericyte coverage increases the success of anti-VEGF treatment in the mouse RIP1-TAG2 model [23]. However, extensive regression of endothelial cells was not observed in tumors after inhibition of PDGFR-β signaling [24]. After treatment of RIP1-TAG-2 tumors and Lewis lung carcinomas with VEGF-Trap, surviving pericytes may become more tightly associated with endothelial cells or have no apparent association with tumor vessels [25]. Treatment of RIP1-TAG2 tumors with anti-PDGFR-β antibody for three weeks reduces pericytes, increases endothelial cell apoptosis but does not seem to reduce tumor vascular density [26]. Treatment with a novel DNA oligonucleotide aptamer (AX102) that selectively binds PDGF-B led to progressive reduction of pericytes in Lewis lung carcinomas [27].

VEGFR-2 blockade can lead to the up-regulation of angiopoietin-1 (Ang-1) that increases pericyte coverage of the vessels [28]. Ang-2 is responsible for blood vessel destabilization in vasculature surrounding tumors. In glioblastoma patients, the Ang-1/Ang-2 ratio correlates with survival [29] and vascular normalization, whereas high Ang-2 levels correlate with resistance to anti-VEGF therapy [30]. Blockade of VEGF signaling with the TKI cediranib significantly reduced levels of Ang-2 in some patients, even if the decrease was transient and modest [30]. Chae et al. expressed Ang-2 in an orthotopic glioma model and demonstrated that ectopic expression of Ang-2 had no effect on vascular permeability, tumor growth, or survival, but it resulted in higher vascular density, with dilated vessels and reduced mural cell coverage [31]. When combined with anti-VEGFR-2 treatment, Ang-2 destabilized vessels and compromised the survival benefit of VEGFR-2 inhibition by increasing vascular permeability, suggesting that VEGFR-2 inhibition normalized tumor vasculature, whereas ectopic expression of Ang-2 diminished the beneficial effects of VEGFR-2 blockade by inhibiting vessel normalization.

The inhibitors of VEGF in the therapy of central nervous system malignancy normalize tumor vasculature and decrease tumor interstitial pressure, leading to an improved access of cyto-reductive drugs and radiotherapy efficacy, due to an increased oxygen delivery [32]. However, these agents may also restore the low permeability characteristics of normal brain microvasculature, counteracting beneficial effects.

## HYPOXIA

Hypoxia mediates immune cells recruitment and these cells concentrate at the tumor periphery, while in the tumor core hypoxia contributes to cancer cell escape by providing an aggressive selection for stem-like tumor cells. Most cancers are hypoxic at the beginning of therapy [33] and hypoxic areas are refractory to chemotherapy and radiotherapy and contribute to select tumor populations able to survive in poorly oxygenated niches and escape to metastatic sites and pro-angiogenic cancer stem cells (CSCs) [34].

VEGF blockade aggravates hypoxia, which up-regulates the production of other angiogenic factors or increases tumor cell invasiveness [14, 35]. Tumor cells respond to hypoxia by becoming tolerant and modifying their metabolic characteristics to resist to low oxygenation [36]. Increased intratumor hypoxia induces the selection of more invasive metastatic clones of the cancer cells that are resistant to anti-angiogenic agents [37], through the production of pro-migratory proteins, such as stromal cell derived factor 1 alpha (SDF1-α) and hepatocyte growth factor- scatter factor (HGF-SF) and pro-invasive extracellular matrix proteins [38, 39]. Hypoxia generated by angiogenesis inhibition triggers pathways that make tumors more aggressive and metastatic and less sensitive to anti-angiogenic treatment, as demonstrated by Paez et al. [35], who used blocking VEGFR-2 antibodies to mouse models of pancreatic neuroendocrine carcinoma and glioblastoma, and found that cancers showed heightened invasiveness or metastasis.

## RECRUITMENT OF INFLAMMATORY CELLS AND IMMATURE MYELOID CELLS

Inflammatory cells act in concert with tumor cells, stromal cells, and endothelial cells to create a microenvironment that is critical for the survival, development, and diffusion of the neoplastic mass. These synergies represent important mechanisms for tumor development and metastasis by providing an efficient vascular supply and an easy escape pathway. The most aggressive human cancers, such as malignant melanoma, breast carcinoma, and colorectal adenocarcinoma, are associated with a dramatic host response composed of various inflammatory cells, including macrophages, mast cells and lymphocytes.

Therapy resistance may be mediated by the recruitment by tumor cells of tumor associated macrophages and mast cells [40], pro-angiogenic bone marrow-derived cells including CD11b^+^ Gr1^+^ myeloid cells [41] and Tie2^+^ monocytes [42], tumor associated fibroblasts (TAFs) [43] and production of alternative pro-angiogenic factors [44], including FGF-2 [45], interleukin-8 (IL-8) [46], IL-17 [47], and ANG-2 [48].

TAFs generated PDGF-C, which is involved in tumor refractoriness to anti-angiogenic therapy, as demonstrated by the use of neutralizing antibodies anti-PDGF-C ameliorate TAF-resistant induced angiogenesis [49]. Moreover, cancer-associated inflammatory cells trigger a catabolic pathway that causes severe adipose and muscular atrophy [50].

A clear involvement of IL-8 has been reported in anti-VEGF tumor resistance in sunitinib-treated renal carcinoma [46]. IL-17 mobilizes the granulocyte-colony stimulating factor (G-CSF)-dependent recruitment of CD11b^+^Gr1^+^ immature myeloid cells, involved in anti-VEGF tumor refractoriness [47]. Human late-stage breast cancers expressed several angiogenic cytokines in contrast to earlier stage lesions, which preferentially expressed only VEGF [51].

## ALTERNATIVE MECHANISMS OF TUMOR VESSEL FORMATION

Other modes of tumor vascularization, including intussusceptive microvascular growth (IMG), vasculogenic mimicry, vascular co-option, differentiation of CSCs into endothelial cells, and vasculogenic vessel growth, might be less sensitive to VEGF blockade.

**Figure 1:**
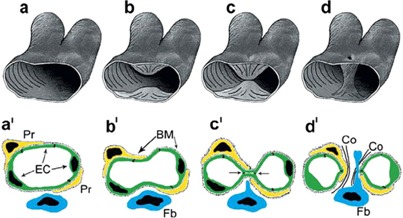
3D (a-d) and 2D (a’-d’) scheme depicting the generation of transluminar pillar by IMG Simultaneously protrusion of opposing capillary walls into the vessel lumen (a,b; a’, b’) results in creation of interendothelial contact zone (c; c’). In a subsequent step the endothelial bilayer becomes perforated and the newly formed pillar core got invaded by fibroblasts (fb) and pericytes (Pr), which lay down collagen fibrils (Co in d’). [Reproduced from 52].

IMG generates vessels more rapidly with a less metabolic demand as compared to sprouting angiogenesis and is a putative strategy that tumors can use for rapid adaptation to milieu changes. No endothelial cell proliferation is required for IMG: endothelial cells only increase their volume and IMG occurs through the splitting of the existing vasculature by transluminal pillars or transendothelial bridges (Figure 1) [52]. Tumors might prefer IMG during anti-angiogenic therapy as it is faster and metabolically more feasible as compared with sprouting angiogenesis [8]. IMG occurs in several tumors, including colon and mammary carcinomas, melanoma, B-cell non Hodgkin's lymphoma, and glioma [53-57]. Treatment of mammary carcinoma allografts with a TKI results in transient reduction in tumor growth rate with decreased tumor vascularization followed by post-therapy relapse with extensive IMG, and the switch to IMG improves the perfusion of the tumor mass [58].

Maniotis et al. [59] described for the first time a new model of formation of vascular channels by human melanoma cells and called it “vasculogenic mimicry” to emphasize the *de novo* generation of blood vessels without the participation of endothelial cells and independent of classical angiogenic factors, including FGF-2 and VEGF [59]. *In vitro* stimulation with VEGF do not enhance vasculogenic mimicry [60] and it has been proposed that vasculogenic mimicry might be dependent by CSCs [61].

In vascular co-option, tumor cells have immediate access to blood vessels, as it occurs in in site of metastases or in densely vascularized organs, including brain, lung, liver, and initiate blood-vessel-dependent tumor growth as opposed to classical angiogenesis. Tumor cells co-opt and growth as cuffs around adjacent vessels [62]. The co-opted vessels initiate an apoptotic cascade mediated by Ang-2 followed by regression of the co-opted vessels. Shortly after regression, hypoxic tumor cells expressing VEGF up-regulate the angiogenic response [62]. Treatment of glioma with a monoclonal antibody anti-VEGFR-2 induces co-option of quiescent cerebral vessels [63] and treatment of cerebral melanoma metastasis with the TKI ZD6474 is associated with increase in vessel co-option [64].

CSCs reside in a vascular niche in close proximity to blood vessels named as CSC niche [65], and generate angiogenic factors to stimulate tumor angiogenesis; tumor vasculature, in turn, supports CSC self-renewal and maintaining. CSCs produce high levels of VEGF in both normal and hypoxic conditions [66]. Moreover, CSCs recruit endothelial precursors for revascularization and tumor re-growth [67, 68].

Ricci-Vitiani et al. demonstrated that *in vitro* culture of glioblastoma stem-like cells in generated a progeny with phenotypic and functional features of endothelial cells [69]. Moreover, orthotopic or subcutaneous injection of glioblastoma stem-like cells in immunocompromised mice generated large anaplastic tumor xenografts, showing a vessel wall formed by human endothelial cells derived from glioblastoma stem-like cells whereas tumor derived endothelial cells formed large anaplastic tumors in secondary recipients [69].

Postnatal vasculogenesis may contribute to tumor vascular supply throughout endothelial precursor cells (EPCs), which circulate from bone marrow, migrate and differentiate in the stromal environment of tumors [70]. High levels of VEGF produced by tumors result in the mobilization of bone marrow-derived EPCs in the peripheral circulation and enhance their recruitment into the tumor vasculature [70].

## GENOMIC INSTABILITY OF TUMOR ENDOTHELIAL CELLS AND REVERSIBILITY OF RESISTANCE

Comprehensive genomic analysis of tumors demonstrates significant genetic intra- and inter-tumor heterogeneity [71]. St Croix et al. [72], were the first to show that colorectal cancer endothelial cells overexpress specific transcripts as a result of qualitative differences in gene profiling compared with endothelial cells of the normal colorectal mucosa. Further studied in glioma [73] and in invasive breast carcinoma [74] demonstrated a distinct gene expression pattern related to extracellular matrix and surface proteins characteristic of proliferating and migrating endothelial cells, and pointed to specific roles for genes in driving tumor angiogenesis and progression of tumor cells. Moreover, endothelial cells isolated from various tumors acquired genotype alterations, leading to altered anti-angiogenic targets and resistance [75], and proximity of tumor cells and endothelial cells within the tumor microenvironment may be responsible for the genotype alterations [76].

Development of a resistance-like phenotype to sorafenib by human hepatocellular carcinoma cells is reversible and can be delayed by metronomic UFT chemotherapy [77]. The continued administration of bevacizumab beyond progression still results in a small significant overall survival [78], suggesting that the resistance if reversible and raising the possibility of re-treating with the same of an alternative VEGF-A inhibitor.

## PREDICTIVE MARKERS

Predictive markers of angiogenesis or anti-angiogenesis are needed to demonstrate the activity and efficacy of anti-angiogenic agents in clinical trials and for the future monitoring of anti-angiogenic treatments in clinics. There are currently no validated biomarkers for selecting patients that benefit from the treatment with anti-angiogenic agents from those patients that will not.

VEGF and VEGF isoforms expression levels serve as a predictive marker for selecting cancer patients who are likely to benefit from anti-VEGF therapy [79]. Elevated levels of sVEGFR-1 prior to treatment were associated with a poor outcome from bevacizumab in rectal carcinoma, hepatocellular carcinoma, and metastatic colorectal carcinoma patients [80, 81]. Increased VEGFR-1 levels may induce increased pro-angiogenic signaling by placental growth factor (PlGF) when VEGF is blocked [79]. Circulating levels of the chemokine SDF1α rise in patients who evade various anti-VEGF therapies including rectal carcinoma with bevacizumab, glioblastoma multiforme with cediraninb, hepatocellular carcinoma with sunitinib, and soft tissue sarcoma with sorafenib [82]. However, measurement of circulating markers is difficult to standardize across different centers due to technical issues associated with sample handling [83].

Medical imaging techniques play an important role in the evaluation of anti-angiogenic treatment efficacy. Dynamic contrast-enhanced perfusion magnetic resonance imaging (MRI), perfusion computed tomography (CT) give only an indirect estimation of angiogenesis. New molecular imaging techniques can give an overall estimation of angiogenesis and anti-angiogenic therapy effects. These include nuclear imaging techniques such as positron emission tomography (PET), that uses paramagnetic nanoparticles to track angiogenesis by targeting avb3 integrin and sonography with novel contrast agents such as gas-filled microbubbles directed against specific target endothelial cell receptors and optical techniques.

## INCREASE OF METASTATIC POTENTIAL

VEGF pathway inhibition may change the natural history of tumor progression after anti-angiogenic therapy and include potential metastasis promoting effects. Short-term treatment with sunitinib prior to intravenous inoculation of breast and melanoma cells could accelerate metastasis and short survival, despite cessation of treatment [84]. Moreover, sunitinib increases metastasis in orthotopic mouse models of breast and colon cancer, whereas it does not promote metastasis in lung cancer [85]. Increased invasiveness might result from enhanced expression of various angiogenic cytokines induced by the treatment, such as VEGF and PlGF, or recruitment of EPCs that promote the formation of a pre-metastatic niche [86]. Hypoxia-driven effects may be also involved, because hypoxia generated by angiogenesis inhibition triggers pathways that make tumors more aggressive and metastatic and less sensitive to anti-angiogenic treatment [35, 84]. Finally, VEGF-targeted therapy can allow an epithelial-mesenchymal transition, which could in turn promote increased invasion and metastasis [87].

## CONCLUSIONS AND PERSPECTIVES

**Figure 2:**
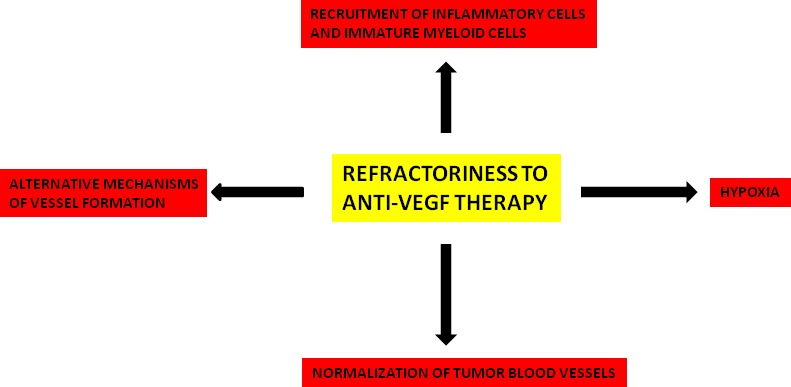
Principal mechanisms involved in refractoriness to anti-VEGF therapy

Mechanisms of resistance can be divided into non-oncogenic and oncogenic, and these latter are associated with highly aggressive cancer phenotype [88]. Anti-angiogenic treatment induces a reactive resistance which is mediated by the HIF/VEGF pathway, allowing both endothelial and cancer cells to resist to therapy [89]. Resistance to VEGF pathway inhibitors involves different mechanisms that are summarized in Figure 2. All of these mechanisms deserve further investigation in both animal models and in humans to clarify their significance and importance.

The successful development of anti-VEGF targeted therapy will require a greater understanding of how tumors become vascularized and how they evade the effects of anti-angiogenic therapy. VEGF blockade aggravates tumor hypoxia, which up-regulates the production of other angiogenic factors in the tumor microenvironment. In this context, targeting VEGF and other pathways implicated in angiogenesis should result in more effective tumor growth inhibition. Moreover, rational combinations of anti-angiogenic agents based on pharmacokinetic and pharmacodynamics data are needed to overcome resistance and it is extremely important to determine the optimal duration and scheduling of anti-VEGF agents. It has been underlined the importance of the time interval of the normalization effects of anti-angiogenesis, the so-called “window of normalization”[90]. Recently, Paez-Ribes et al. [91] have demonstrated that metastatic effects of preclinical anti-angiogenic therapy with an antibody targeting mouse VEGFR-2 are prevented by concurrent chemotherapy.

The identification of specific predictive biomarkers (Table 2) remains an important end-point even if biomarkers that are predictive of anti-VEGF therapy may be specific to different tissues and tumor subtypes.

**Table 2:** Biomarkers to predict response to anti-VEGF inhibitors

Functional imaging
Hypertension
Circulating proteins (baseline plasma VEGF concentration and soluble VEGFR-2)
Circulating endothelial or tumor cells
Single nucleotide polymorphisms (SNPs)
Tumor biomarkers (CD31-positive tumor vessels; Tumor neuropilin 1 immunoreactivity; plasma levels of intercellular adhesion molecule-1)
